# Whole-body transcriptome of selectively bred, resistant-, control-, and susceptible-line rainbow trout following experimental challenge with *Flavobacterium psychrophilum*

**DOI:** 10.3389/fgene.2014.00453

**Published:** 2015-01-08

**Authors:** David Marancik, Guangtu Gao, Bam Paneru, Hao Ma, Alvaro G. Hernandez, Mohamed Salem, Jianbo Yao, Yniv Palti, Gregory D. Wiens

**Affiliations:** ^1^National Center for Cool and Cold Water Aquaculture, Agricultural Research Service, United States Department of AgricultureKearneysville, WV, USA; ^2^Department of Biology, Middle Tennessee State UniversityMurfreesboro, TN, USA; ^3^Animal and Nutritional Sciences, West Virginia UniversityMorgantown, WV, USA; ^4^High-Throughput Sequencing and Genotyping Unit, Roy J. Carver Biotechnology Center, University of Illinois at Urbana-ChampaignUrbana, IL, USA

**Keywords:** *Flavobacterium psychrophilum*, bacterial cold water disease, selective breeding, disease resistance, aquaculture, immune gene, tnfrsf, rainbow trout genome

## Abstract

Genetic improvement for enhanced disease resistance in fish is an increasingly utilized approach to mitigate endemic infectious disease in aquaculture. In domesticated salmonid populations, large phenotypic variation in disease resistance has been identified but the genetic basis for altered responsiveness remains unclear. We previously reported three generations of selection and phenotypic validation of a bacterial cold water disease (BCWD) resistant line of rainbow trout, designated ARS-Fp-R. This line has higher survival after infection by either standardized laboratory challenge or natural challenge as compared to two reference lines, designated ARS-Fp-C (control) and ARS-Fp-S (susceptible). In this study, we utilized 1.1 g fry from the three genetic lines and performed RNA-seq to measure transcript abundance from the whole body of naive and *Flavobacterium psychrophilum* infected fish at day 1 (early time-point) and at day 5 post-challenge (onset of mortality). Sequences from 24 libraries were mapped onto the rainbow trout genome reference transcriptome of 46,585 predicted protein coding mRNAs that included 2633 putative immune-relevant gene transcripts. A total of 1884 genes (4.0% genome) exhibited differential transcript abundance between infected and mock-challenged fish (FDR < 0.05) that included chemokines, complement components, tnf receptor superfamily members, interleukins, nod-like receptor family members, and genes involved in metabolism and wound healing. The largest number of differentially expressed genes occurred on day 5 post-infection between naive and challenged ARS-Fp-S line fish correlating with high bacterial load. After excluding the effect of infection, we identified 21 differentially expressed genes between the three genetic lines. In summary, these data indicate global transcriptome differences between genetic lines of naive animals as well as differentially regulated transcriptional responses to infection.

## Introduction

Selective breeding programs contribute to increased aquaculture production through the generation of animals with improved resistance/tolerance toward infectious disease causing microorganisms (Gjedrem, 1983, 2005; Van Muiswinkel et al., 1999; Cock et al., 2009; Gjedrem et al., 2012). In 2005, a family-based selective breeding program was initiated at the National Center for Cool and Cold Water Aquaculture (NCCCWA) to improve rainbow trout (*Oncorhynchus mykiss*) survival following exposure to *Flavobacterium psychrophilum* (Silverstein et al., 2009). This pathogen is the etiologic agent of bacterial cold water disease (BCWD) and rainbow trout fry syndrome (RTFS), and causes considerable losses to the rainbow trout aquaculture industry within the U.S. and to trout and salmon populations worldwide (Nematollahi et al., 2003; Barnes and Brown, 2011). Infection of rainbow trout with *F. psychrophilum* typically results in mortality, ranging from 2 to 30% of the population, with higher losses caused by co-infection with the infectious hematopoietic virus. A further impact of the disease is that survival following infection has been associated with skeletal deformities (Kent et al., 1989; Madsen et al., 2001). Disease prevention is difficult as the pathogen is geographically wide spread, limited chemotherapeutants are available for treatment, and there is currently no commercial vaccine available in the U.S, although killed, subunit, and live-attenuated vaccines are all active areas of research (Gómez et al., 2014; Sundell et al., 2014).

A pedigreed line of rainbow trout, designated ARS-Fp-R, has been subjected to over three generations of selection and demonstrates increased survival following experimental injection challenge (Hadidi et al., 2008; Silverstein et al., 2009; Leeds et al., 2010) and natural exposure (Wiens et al., 2013a), relative to a disease susceptible line, ARS-Fp-S, and a randomly bred control line, ARS-Fp-C. Current research goals at the NCCCWA includes elucidating intrinsic factors associated with survival of the ARS-Fp-R line to better understand mechanisms of how selection has altered the genetic control of disease resistance. Phenotypic studies have thus far shown that ARS-Fp-R line fish have decreased organ damage as determined by histopathology (Marancik et al., 2014b) and fewer pathophysiologic changes in plasma biochemistry (Marancik et al., 2014a) during the acute-phase of disease following experimental challenge. Experiments that quantified splenic *F. psychrophilum* numbers on days 5 (Hadidi et al., 2008) and 9 post-infection (Marancik et al., 2014a) demonstrate significantly lower splenic bacterial loads in ARS-Fp-R line fish. It is likely these observed differences are a result of a differential immune response to infection.

Changes associated with host immunologic response can be elucidated by profiling alterations in host mRNA abundance between pathogen naive and infected animals (Martin et al., 2006; Beck et al., 2012; Langevin et al., 2012; Peatman et al., 2013; Pereiro et al., 2014; Shi et al., 2014). Previous studies of rainbow trout infected with *F. psychrophilum* demonstrate significant upregulation and downregulation of rainbow trout immune-relevant genes in the limited number of tissues examined (Overturf and LaPatra, 2006; Villarroel et al., 2008, 2009; Evenhuis and Cleveland, 2012; Langevin et al., 2012; Orieux et al., 2013; Henriksen et al., 2014). Microarray analysis of head kidney tissue from susceptible compared to resistant double-haploid rainbow trout lines, identified differences in basal gene expression as well as induction of antimicrobial peptides, complement, matrix metalloproteases, and chemokines 5 days post-infection (Langevin et al., 2012). Taken together, these studies suggest that the rainbow trout immune response to *F. psychrophilum* is likely multifactorial involving both innate and adaptive components.

In this manuscript, we quantify changes in gene transcript abundance between genetic lines with the following goals: (1) identify differentially regulated genes common to the host response to *F. psychrophilum* infection, (2) identify genes differentially regulated between lines in response to *F. psychrophilum* infection, and finally, (3) examine baseline differences in gene expression between naive animals that might contribute to the post-challenge phenotype. For this study, we utilized a comprehensive RNA-seq, transcriptome approach, starting with whole-body lysates from 1.1 g fry from pooled fish of naive or experimentally infected genetic lines. Experimental infection during the fry/juvenile life-stage allowed gene transcript profiling at a time when genetic lines express robust survival differences, and when epizootics and mortality are described as most severe in production environments (Branson, 1995; Decostere et al., 2000). Sequence reads were aligned to the recently released rainbow trout genome transcriptome (Berthelot et al., 2014) to which we added automated annotation and manual immune gene curation. In summary, we described common, whole-body gene transcriptional responses to early *F. psychrophilum* infection and potential differences associated with the innate response between genetic lines, and finally, compare our results with published gene expression studies utilizing *F. psychrophilum* challenged rainbow trout.

## Materials and methods

### Ethic statement

Fish were maintained at the NCCCWA and animal procedures were performed under the guidelines of NCCCWA Institutional Animal Care and Use Committee Protocols #053 and #076.

### Experimental animals

The ARS-Fp-R, ARS-Fp-C, and ARS-Fp-S genetic lines were derived from the same base population, and thus differed only as a result of artificial selection for BCWD post-challenge survival (Wiens et al., 2013a). Single-sire × single-dam matings were made within genetic lines between 3-year-old females and 1-year-old neo-males as previously described (Marancik et al., 2014b). Water temperatures in the egg incubation jars were manipulated so that all families hatched within a 1-week period (Leeds et al., 2010). Eggs were pooled within-line at the eyed stage and reared in ~12.5°C flow-through spring water. The ARS-Fp-R egg pool consisted of contributions from 43 full-sib families, the ARS-Fp-C egg pool consisted of 10 full-sib families, and the ARS-Fp-S egg pool consisted of 11 full-sib families. The resistant line eggs were progeny of dams that had undergone three generations of BCWD selection while the sires had undergone four generations of selection. The control-line eggs were progeny of parents that had undergone one generation of selection for increased resistance (2007 year class) and since that time, randomly bred. The susceptible-line eggs were progeny of parents that had undergone one generation of selection for increased susceptibility (2007 year class) and since that time, randomly bred (Wiens et al., 2013a). Eggs were pooled from a larger number of resistant line families as more resistant families are generated within the breeding program to apply selection differential, and thus this sampling design more accurately captured the genetic diversity within the resistant line. In addition, the resistant-line egg pool was part of a germ-plasm release and was utilized in additional challenge studies that will be reported elsewhere (Wiens, unpublished data).

All brood-stock and fish used in this study were certified to be free of common salmonid bacterial and viral pathogens by two independent diagnostic laboratories as described previously, and were negative for *F. psychrophilum* infection (Leeds et al., 2010; Wiens et al., 2013a). Prior to challenge, fry were allowed 1 week to acclimatize to challenge tanks. Mean body weight of the ARS-Fp-R line fish was 1.11 ± 0.05 g, the ARS-Fp-C line was 1.12 ± 0.03 g, and the ARS-Fp-S line was 0.98 ± 0.04 g (±1 SD, pooled weights of *n* = 4 tanks) at the initiation of the challenge. In the challenge facility, photoperiod was adjusted weekly to maintain a natural lighting cycle, and at the time of RNA-seq sample collection, was 14.5 h light: 9.5 h dark. Water quality parameters have been described previously (Wiens et al., 2013a).

### RNA-seq experimental design

Bacterial challenge was carried out in the NCCCWA challenge facility with *F. psychrophilum* strain CSF-259-93 (initially provided by Dr. S. LaPatra, Clear Springs Foods, Inc.). This strain was previously isolated from a BCWD field-case and maintained at −80°C in TYES media supplemented with 10% (v/v) glycerol and has been consistently utilized as the challenge strain within the selective breeding program (Hadidi et al., 2008; Silverstein et al., 2009; Leeds et al., 2010; Wiens et al., 2013a) and the complete genome sequence determined (Wiens et al., 2014). Frozen stock was cultivated on TYES media for 5 days at 15°C, suspended in PBS and O.D._525_ adjusted to 0.4. Colony plate counts were performed in triplicate and recorded after 5 days incubation to estimate the challenge dose enumerated as viable CFU fish^−1^.

Each tank held fifty, randomly assigned fish and were supplied with 2.4 L min^−1^ of 12.5 ± 0.1 °C flow-through spring water. For each genetic line, two tanks of fish were challenged by *F. psychrophilum* injection and two tanks of fish were challenged by PBS injection and served as non-infected control animals. In total, 100 fish per line were anesthetized with 100 mg/L tricaine methanesulfonate (Tricaine-S, Western Chemical, Inc., Ferndale, WA) and intraperitoneally (IP) injected with 4.2 × 10^6^ CFU fish^−1^ *F. psychrophilum* suspended in 10 μL of chilled PBS or 10 μL of chilled PBS alone. Injections were performed using a repeater pipette (Eppendorf, Hauppauge, NY) fitted with a 27G × 1/2 inch needle. Fish age at the time of challenge was 49 days post-hatch (617 temperature degree days).

Five fish were sampled per tank on day 1 and on day 5 post-injection for RNA extraction. Survival prior to and following sampling was monitored daily for 21 days with the exception of one PBS injected tank that was excluded on day 16 due to water failure. All fish were fed daily a standard commercial fishmeal-based diet by hand (Ziegler Bros, Inc., USA). The day 1 sampled fish were removed prior to being fed and the day 5 sampled fish were removed after being fed.

### RNA extraction, library preparation, and sequencing

The sampled fish were euthanized with 150 mg mL^−1^ tricaine methanesulfonate and individually flash frozen in liquid nitrogen and stored at −80°C. Total RNA was extracted from individual whole, ground fish using the standard TRIzol protocol (Invitrogen, Carlsbad, CA). Total RNA was extracted and integrity confirmed by running a 1% agarose gel. Equal amounts of RNA from five fish were pooled from each of the 12 tanks at each of the two time points (total of 24 pools, *n* = 120 fish total). The cDNA libraries were prepared using Illumina's TruSeq Stranded mRNA Sample Prep kit following the manufacturer's instructions. Briefly, mRNA was selected with oligo(dT) beads and chemically fragmented to a size of ~100–400 nt before annealing of random hexamers and first strand cDNA synthesis. The 24 indexed and barcoded libraries were randomly divided into three groups (eight libraries per group) and sequenced in three lanes of an Illumina HiSeq 2000 (single-end, 100 bp read length) at the University of Illinois at Urbana-Champaign. All raw RNA-seq reads were submitted to the NCBI Short Read Archive under accession number BioProject ID PRJNA259860 (accession number SRP047070). RNA sequence reads that matched the *F. psychrophilum* CSF259-93 genome sequence (GenBank accession CP007627.1) were separated from host RNA-seq data and counted in each library.

Frozen fish homogenate lysates (500 μL), stored at −80°C, were individually processed to isolate genomic DNA (TRIzol DNA Isolation Procedure) and qPCR detection of *F. psychrophilum* genomic DNA was performed as described previously (Marancik and Wiens, 2013). Bacterial genome equivalents were normalized to per 100 ng^−1^ extracted DNA.

### Genes described as regulated in response to *F. psychrophilum* challenge

Based on a meta-analysis of studies in which rainbow trout were challenged with *F. psychrophilum*, 23 genes encoding immune relevant factors with putative roles in inflammation, innate disease response, and adaptive immunity were utilized as a curated gene reference set that included *cd3, cd8, mx-1* (Overturf and LaPatra, 2006), *saa* (Villarroel et al., 2008), *igm, igt, inf*-*γ, il-8, tcr-β, tlr5, tnf-α* (Evenhuis and Cleveland, 2012), *mt-a, sod-1, tgf-β* (Orieux et al., 2013), and *cd4, il-1β, il-6, il-17c1, il-17c2, IL-4/13A, foxp3b, mhc-I*, and *mhc-II* (Henriksen et al., 2014).

### Rainbow trout reference genome, GO annotation, and identification of immune relevant genes

In addition to the curated reference gene dataset described above, the recently released rainbow trout genome sequence accession CCAF000000000 (Berthelot et al., 2014) encoding 46,585 predicted coding mRNA sequences were utilized as a more complete reference set of gene models. Briefly, the Onchorhynchus_mykiss_pep.fa file (April 24, 2014 release), accessed at (http://www.genoscope.cns.fr/trout-ggb/data), was subjected to Blast2GO sequence annotation pipeline with the default parameters applied in the blast, mapping, and annotation steps using a local Blast2GO database (GO database file go_201401-assocdb-data). NCBI non-redundant protein sequence (nr) database (build 12/13/2013) was used as the reference in the blast step. Of the 46,585 protein-coding gene models with supporting protein evidence from other vertebrates, a total of 46,103 were assigned a “Sequence Description” from the blast step and most of these predicted protein sequences assigned GO terms (Supplementary Data Sheet 1) from the Blast2GO annotation step. A subset of 2633 genes were identified as “putative immune relevant” either by sorting genes identified by GO annotation as “Immune Response” or by manual annotation based on sequence description (Supplementary Data Sheet 2). We excluded from analyses gene models encoding long non-coding RNAs and microRNAs. Splice variants were not analyzed and will be the subject of further study.

### Identification of differentially expressed genes

To identify the differentially expressed genes, reads from each library were mapped against the annotated gene database and the coding sequences from the rainbow trout genome assembly. Based on the high quality score distribution of the RNA-seq reads, the whole 100 bp of the sequences were used in this step. Bowtie short read aligner (Langmead et al., 2009) was used in mapping the reads to the references with a maximum of two mismatches allowed and no gaps. The output of Bowtie was filtered with an in-house Perl script to generate a count table, where a number at the corner of row i and column j represents the number of total mapped reads to the transcript i from the library j. For principle component analyses (PCA), reads were normalized to reads per kilobase of exon model per million mapped reads (RPKM) using an in-house script (Gao, available upon request) using the formula described previously (Mortazavi et al., 2008).

$$\begin{array}{l} RPKM=N_{read}/\left(L_{exon}/10^{3}\right)/\left(N_{total}/10^{6}\right)\\ \;=10^{9}N_{read}/\left(L_{exon}N_{total}\right) \end{array}$$

Where *N_read_* is defined as the number of reads mapped to the gene; *L_exon_* is defined as the total bases of the sequence of the gene, and *N_total_* is defined as the total number of reads mapped to the whole reference (sum of the *N_read_* for all genes). Raw data and normalized RNA-seq data (*RPKM*) for all samples including immune relevant genes are included as Supplementary Data (Supplementary Data Sheet 3).

Principal component and nearest neighbor network analyses were performed using Qlucore Omics Explorer (v3.0). Read count data from the 24 samples (RPKM) were *log*_2_ transformed and subjected to normalization (mean = 0 and variance = 1) and variables (genes) subjected to multi-group comparison with a false discovery rate (FDR) *q* < 0.05 (*R*^2^ ≥ 0.36) and *p* < 0.002 [*F*_(1, 22)_ ≥ 12.42].

The raw count table was also input into the R package DESeq2 (Love et al., 2014), datasets selected for pair-wise comparisons, and the standard differential analysis steps of DESeq2 applied to the selected datasets. The output table of DESeq2 contains a column of adjusted *p*-value (padj) obtained using the Benjamini-Hochberg procedure (Benjamini and Hochberg, 1995), and we utilized a cut-off of *p_adj_* < 0.05 as a criteria for differential expression with no filtering based on fold-change. For more stringent data filtering and visualization, data were first sorted by variance 2.5%, then filtered by *q* < 0.01 and *log*_2_ fold change of >3 (Qlucore Omics Explorer v3.0).

### GO annotation enrichment analyses

The GOSSIP program embedded in the Blast2GO package was used for GO enrichment analysis (Bluthgen et al., 2005a). This program examines each GO term for gene annotation enrichment by comparing a test set with a reference set using Fisher's exact-test method. In our analysis, the differentially expressed genes were selected as the test set and all the peptide sequences from the rainbow trout genome assembly for which GO terms were assigned, excluding the test set, were used as the reference set. FDR is computed by GOSSIP using an analytical equation (Bluthgen et al., 2005b) and the final list of the enriched GO terms selected at the FDR < 5%.

### Validation of RNA-seq data by qPCR

Six genes and primer sets were chosen for qPCR analysis to determine fold-change differences between control and infected fish on day 5 post-infection (Table S1). Extracted RNAs were treated with Optimize™ DNAase I (Fisher Bio Reagents, Hudson, NH) to remove contaminating genomic DNA. One microgram of DNAase treated RNA was used to make cDNA in a total reaction volume of 20 μL. cDNA was synthesized using the Verso cDNA Synthesis Kit (Thermo Scientific, Hudson, NH) following the manufacturer described protocol. Reverse transcription reaction was carried out using My Cycler™ Thermal Cycler (Bio Rad, Hercules, CA) at 42°C for 30 min (one cycle amplification) followed by 95°C for 2 min (inactivation). Anchored oligo(dT) primer, at a final concentration of 25 ng/μL, was used to prime the reverse transcription reaction.

Relative gene expression was determined by CFX96™ Real Time System (Bio Rad, Hercules, CA). Forward and reverse primer sequences were manually aligned to their respective genes for validation. cDNA amplification was performed using DyNAmo Flash SYBR Green Master Mix (Thermo Scientific, Hudson, NH) containing 0.1 nm/μL forward and reverse primers and 0.0025 μg/μL of cDNA template in a total reaction volume of 20 μL. Initial denaturation was done at 95°C for 7 min. Forty-three cycles of amplification were carried out at the condition: 95°C for 0.1 min (denaturation), 57 −64°C (annealing and extension) and 60°C for 5 min (final extension). Different annealing temperatures were used for different primers depending on their melting temperature (Table S1).

Real time data were analyzed using the software Bio-Rad CFX Manager (Bio Rad, Hercules, CA). Differential gene expression was calculated using the standard curve method in which β-actin (Accession: AJ438158) was used as endogenous reference to normalize the target gene. β-actin expression levels demonstrated in RNA-seq data were similar in PBS and *F. psychrophilum*-injected fish (data not shown). qPCR data were quantified using delta delta Ct (ΔΔCt) methods (Schmittgen and Livak, 2008). Ct-values of β-actin were subtracted from Ct-values of the target gene to calculate the normalized value (ΔCt) of the target gene in both the calibrator samples (PBS-injected) and test samples (*F. psychrophilum*-injected). The ΔCt value of the calibrator sample was subtracted from the ΔCt value of the test sample to get the ΔΔCt value. Fold change in gene expression in the test sample relative to the calibrator sample was calculated by the formula 2^−ΔΔCt^ and the normalized target Ct values in each infected and non-infected group was averaged. Correlation between gene expression fold-change measured by qPCR and RNA-seq was performed by Pearson correlation. All statistics were performed with a significance of *P* < 0.05.

## Results

### Disease resistance phenotype and *F. psychrophilum* load in three genetic lines following experimental challenge

**Figure 1:**
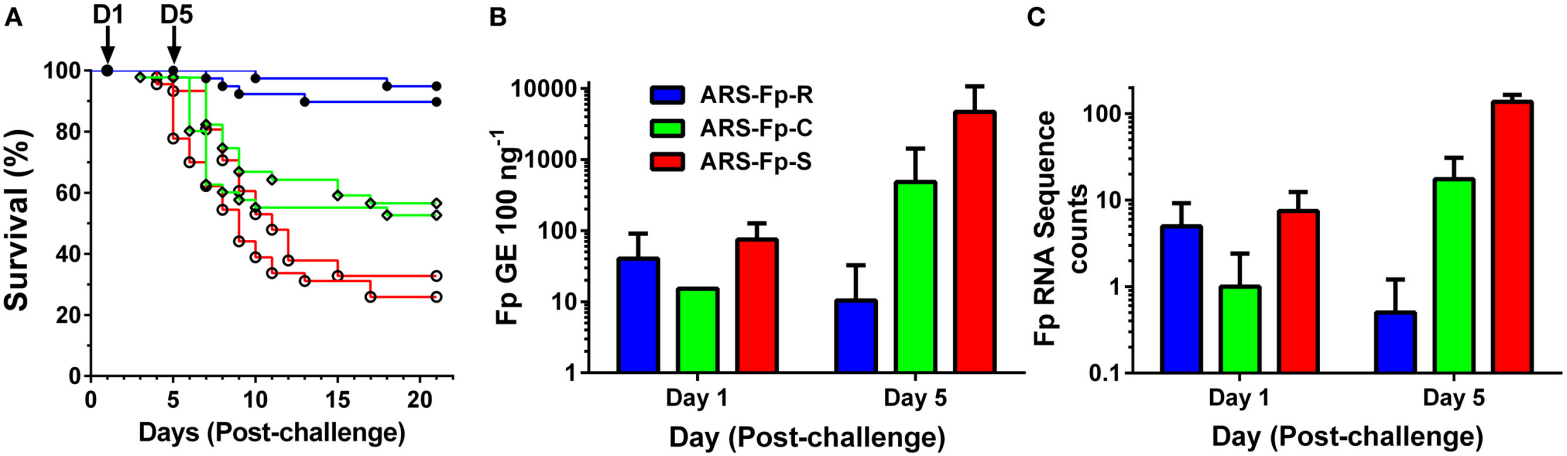
**(A)** Post-challenge survival of ARS-Fp-R line (resistant, blue line), ARS-Fp-C line (control, green line), and ARS-Fp-S line (susceptible, red line) fish, challenged using replicate tanks. Survival differences were significant between genetic lines (*P* < 0.001). Fish were injected with either *F. psychrophilum* in PBS (*n* = 100 fish per line) or with PBS alone (data not shown) and survival monitored for 21 days. Five fish were sampled from each tank (*n* = 10 total) on days 1 and 5 post-challenge (arrows) for RNA-seq analysis. **(B)** Mean *F. psychrophilum* load, genome equivalents per 100 ng extracted DNA (+1 SD) measured by qPCR. Individual fish were tested (*n* = 10 fish per group) with the exception of day 1 ARS-Fp-C line (*n* = 1) as samples were not available. Load differences were significantly different between genetic lines on day 5 (*P* < 0.001). **(C)** Mean *F. psychrophilum* cDNA count per library (+1 SD) identified from the RNA-seq dataset.

Following injection challenge, survival significantly differed between the three genetic lines (*P* < 0.001) with the ARS-Fp-R line exhibiting highest survival (92.3%), the ARS-Fp-C line intermediate survival (54.6%), and the ARS-Fp-S line exhibiting the lowest survival (29.4%) (Figure 1A). The 62.9 percentage point survival difference between the ARS-Fp-R and ARS-Fp-S lines is consistent with the expected percentage point survival difference calculated from estimated parental breeding values (data not shown), analyzed as previously described (Leeds et al., 2010). Survival of PBS injected fish was >98% per line over the 21 days challenge period. Whole body *Fp* loads, measured by qPCR, demonstrated no difference between genetic lines on day 1 but significant differences on day 5 (*P* < 0.001), with ARS-Fp-R line mean loads of 10 ± 22 *F. psychrophilum* genome equivalents (GE) (*n* = 10 fish, ±1 SD), increasing to 484 ± 937 GE in the ARS-Fp-C line (*n* = 10, ±1 SD), and highest mean load was present in the ARS-Fp-S line 4683 ± 6011 GE (*n* = 10, ±1 SD) (Figure 1B). In the RNA-seq libraries, a small number of sequences were present that matched the *F. psychrophilum* CSF259-93 genome. These presumably represent either cDNA from bacterial expressed genes or contaminating genomic DNA. The abundance of the *F. psychrophilum* read counts were similar between genetic lines on day 1, and by day 5, lowest in ARS-Fp-R line fish and highest in ARS-Fp-S line fish (Figure 1C). In summary, by day 5 post-challenge the phenotypes of the ARS-Fp-R, ARS-Fp-C and ARS-Fp-S lines displayed expected differences in pathogen load and subsequently post-challenge mortality.

### Rainbow trout RNA-seq reference dataset, GO annotation, and manual immune gene curation

A total of 520 million sequence reads were generated from the 24 libraries with an average of 21.7 million (M) RNA-seq reads per library ranging from 17.4 M to 24.4 M reads (Table 1). Approximately half of the reads aligned to the reference transcriptome coding sequence, averaging 11.2 M (51.8%) per library with a range of 9.3 M–12.5 M. Of the 46,585 genes identified in the rainbow trout genome having protein coding evidence, an average of 43,068 (92.4%), exhibited detectable levels of expression, defined as >1 sequence read per gene (Table 1). The number of genes with an average of ≥10 read counts across the 24 libraries fell to an average of 32,830 (70.5%). In order to normalize gene expression across samples, data were converted to reads per kilobase of exon model per million mapped reads (RPKM) format (Supplementary Data Sheet 3). Of the putative immune relevant genes, 1797 (68.2%) had an average of ≥1 RPKM across the 24 libraries (Supplementary Data Sheet 3).

**Table 1:** **Summary statistics of 24 RNA-seq libraries listed by genetic line, time, infection status, and tank replicate**.

**Genetic line**	**Day**	**Infection, Tank**	**Biosample Accession No**	**Total reads**	**Mapped reads**	**Percentage reads mapped**	**Uniquely mapped reads**	**Number of expressed genes^a^**
ARS-Fp-R	1	Fp, Tk25	SAMN03014722	20,061,852	10,210,344	50.89%	9,552,696	42,919
		Fp, Tk26	SAMN03014723	20,226,280	10,591,198	52.36%	9,963,273	42,044
	5	Fp, Tk25	SAMN03014726	21,409,329	10,973,051	51.25%	10,445,614	43,000
		Fp, Tk26	SAMN03014727	23,958,681	11,795,961	49.23%	11,135,354	43,654
	1	PBS, Tk27	SAMN03014724	22,129,914	11,745,879	53.08%	10,998,947	43,303
		PBS, Tk28	SAMN03014725	23,909,110	12,294,973	51.42%	11,410,381	43,567
	5	PBS, Tk27	SAMN03014728	24,361,298	12,124,063	49.77%	11,507,535	43,446
		PBS, Tk28	SAMN03014729	23,318,224	11,741,589	50.35%	11,171,392	43,504
ARS-Fp-C	1	Fp, Tk33	SAMN03014738	20,940,097	11,050,478	52.77%	10,225,748	43,229
		Fp, Tk34	SAMN03014739	19,151,755	10,393,600	54.27%	9,629,194	42,162
	5	Fp, Tk33	SAMN03014742	21,117,398	10,817,377	51.22%	10,299,653	42,904
		Fp, Tk34	SAMN03014743	21,763,498	10,846,416	49.84%	10,311,809	43,308
	1	PBS, Tk35	SAMN03014740	20,314,994	11,045,087	54.37%	10,314,237	41,735
		PBS, Tk36	SAMN03014741	20,372,060	10,581,232	51.94%	9,871,584	43,173
	5	PBS, Tk35	SAMN03014744	24,480,995	12,593,510	51.44%	11,896,143	43,345
		PBS, Tk36	SAMN03014745	20,535,582	10,422,413	50.75%	9,824,416	43,013
ARS-Fp-S	1	Fp, Tk29	SAMN03014730	17,389,164	9,377,592	53.93%	8,617,417	42,438
		Fp, Tk30	SAMN03014731	21,095,859	11,103,652	52.63%	10,198,695	42,765
	5	Fp, Tk29	SAMN03014734	21,467,982	11,073,977	51.58%	10,409,769	43,263
		Fp, Tk30	SAMN03014735	23,427,268	11,922,324	50.89%	11,172,000	43,479
	1	PBS, Tk31	SAMN03014732	20,622,290	11,310,300	54.85%	10,482,544	42,903
		PBS, Tk32	SAMN03014733	24,139,642	12,522,402	51.87%	11,594,067	43,561
	5	PBS, Tk31	SAMN03014736	22,371,928	11,319,845	50.60%	10,753,477	43,607
		PBS, Tk32	SAMN03014737	22,338,190	11,425,396	51.15%	10,750,682	43,319
Average				21,704,308	11,220,111	51.77%	10,522,359	43,068

*Total number of 100 bp reads and the number that match reference genes are listed*.

a *The number of expressed genes is defined as genes having at least one read count with 2 bp or less mismatch and no gaps (n = 46,585 reference genes)*.

### Global transcript abundance differences between control and infected and by sample day

**Figure 2:**
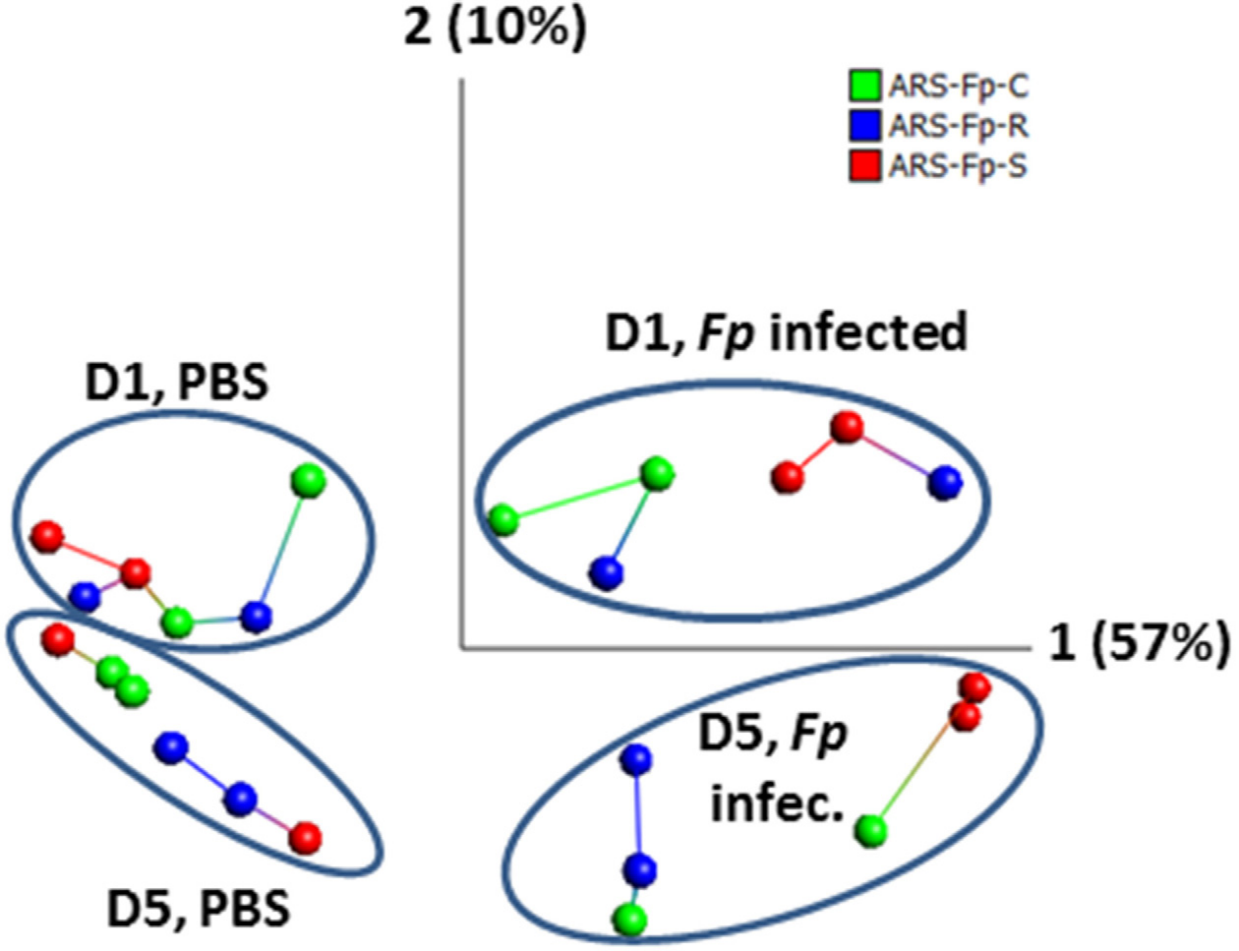
**Principal component analysis of 24 RNA-seq libraries analyzed by infection status**. Across the entire dataset, a total of 1884/46,585 (4.04%) genes were differentially expressed and included within the PCA analysis (false discovery rate *q* = 0.05, *p* = 0.002, no fold-change criteria). Each point represents a single RNA-seq library color coded by genetic line. Samples cluster by day and infection. Lines connecting samples represent the results from nearest neighbor analysis calculated using Qlucore Omics Explorer (v3.0). RPKM data were *log*_2_ normalized. PCA1 accounts for 57% of the variation while PCA2 accounts for 10%.

Across the complete dataset, comparison of *F. psychrophilum* infected vs. PBS injected groups by principal component and nearest neighbor network analysis identified samples grouped by day and infection status (Figure 2). Most tank replicates were connected by nearest-neighbor analysis although there was variation observed between tanks. The replicate tanks for the day 5 PBS injected ARS-Fp-S line (red colored balls), day 1 *F. psychrophilum* infected ARS-Fp-R line (blue colored balls), and day 5 *F. psychrophilum* ARS-Fp-C line (green colored balls) grouped together but were not directly connected by the network analysis.

### Differentially expressed genes common to the three genetic lines in response to *F. psychrophilum* infection

Two-group comparison by infection (Qlucore) identified a total of 1884 genes as differentially regulated (*q* < 0.05) accounting for approximately 4.04% of the coding genes identified in the genome (see Table S2, **Qlucore *q* < 0.05** for the complete list). Of the differentially regulated genes, 279 (14.8%) were categorized as immune relevant by GO or manual annotation. In order to more precisely identify differences between samples, 24 pair-wise comparisons were tabulated by DESeq2 using a *q* < 0.05 (Table 2). There was a decrease in the number of differentially regulated genes in the infected ARS-Fp-R line fish compared to PBS injected fish between day 1 (*n* = 515 genes) and day 5 (*n* = 428 genes) time points. In contrast, the number of differentially regulated genes in the infected ARS-Fp-C line fish compared to PBS injected fish, increased from day 1 (*n* = 20 genes) to day 5 (*n* = 2201 genes). The number of differentially regulated genes in the infected ARS-Fp-S line fish compared to PBS injected fish increased from day 1 (*n* = 1663) to day 5 (*n* = 2225). In general, there were relatively few differentially regulated genes between the three PBS injected genetic lines, ranging from 3 to 246 genes (Table 2). Bacterial challenge increased the number of differentially expressed genes between genetic lines from day 1 to day 5 in all between-line comparisons. There was a strong sampling time effect within each line including PBS injected groups possibly due to differences in feeding or post-injection recovery.

**Table 2:** **Summary of pairwise comparison between treatment groups including infection status, genetic line, and day (*q* ≤ 0.05)**.

**Comparisons**	**Day, Genetic line, and Infection status^a^**	**Diff. expressed genes**
Infected vs. PBS	Day1 R-line (Fp) vs. R-line (PBS)	515
	Day5 R-line (Fp) vs. R-line (PBS)	428
	Day1 C-line (Fp) vs. C-line (PBS)	20
	Day5 C-line (Fp) vs. C-line (PBS)	2201
	Day1 S-line (Fp) vs. S-line (PBS)	1663
	Day5 S-line (Fp) vs. S-line (PBS)	2225
Genetic lines-PBS	Day1 R-line (PBS) vs. S-line (PBS)	76
	Day1 R-line (PBS) vs. C-line (PBS)	3
	Day1 S-line (PBS) vs. C-line (PBS)	28
	Day5 R-line (PBS) vs. S-line (PBS)	45
	Day5 R-line (PBS) vs. C-line (PBS)	246
	Day5 S-line (PBS) vs. C-line (PBS)	61
Genetic lines-*Fp Inf*.	Day1 R-line (Fp) vs. S-line (Fp)	150
	Day5 R-line (Fp) vs. S-line (Fp)	1016
	Day1 R-line (Fp) vs. C-line (Fp)	28
	Day5 R-line (Fp) vs. C-line (Fp)	159
	Day1 S-line (Fp) vs. C-line (Fp)	37
	Day5 S-line (Fp) vs. C-line (Fp)	1758
Between time points	Day5 vs. Day1 R-line (PBS)	1286
	Day5 vs. Day1 C-line (PBS)	294
	Day5 vs. Day1 S-line (PBS)	376
	Day5 vs. Day1 R-line (Fp)	334
	Day5 vs. Day1 C-line (Fp)	2469
	Day5 vs. Day1 S-line (Fp)	2434

a *Abbreviations: R, ARS-Fp-R; C, ARS-Fp-C; S, ARS-Fp-S; Fp, F. psychrophilum challenged; PBS, phosphate buffered saline injected*.

Analysis of GO term enrichment within the pair-wise comparisons revealed a larger number of over-represented terms within the dataset as compared to under-represented terms (Table 3). Overrepresented processes in the ARS-Fp-S line included defense response to bacterium, inflammatory response, leukotriene and arachidonic acid production, complement activation, humoral immune response, antigen processing, chemotaxis, B cell homeostasis, interleukin-2 mediated pathway signaling and cellular iron homeostasis (Supplementary Data Sheet 5 for complete list). The ARS-Fp-R line Day 1 GO term enrichment included several categories that include response to wounding and wound healing and was enriched for cytokine and chemokine activity. ARS-Fp-R line day 5 GO term enrichment included complement activation, inflammatory response, B cell mediated immunity and defense response to bacterium.

**Table 3:** **GO enrichment categories determined from pair-wise comparison**.

**Pair-wise comparison^a^**	**Total**	**Over-represented**	**Under-represented**
GORich_Fp_PBS_D1_R	53	50	3
GORich_Fp_PBS_D1_C	11	11	0
GORich_Fp_PBS_D1_S	361	218	143
GORich_Fp_PBS_D5_R	326	156	170
GORich_Fp_PBS_D5_C	909	611	298
GORich_Fp_PBS_D5_S	607	333	274

a *Abbreviations: Fp, F. psychrophilum challenged vs. PBS, phosphate buffered saline injected; D1, day1; D5, day5; R, ARS-Fp-R line; C, ARS-Fp-C line; S, ARS-Fp-S line*.

**Figure 3:**
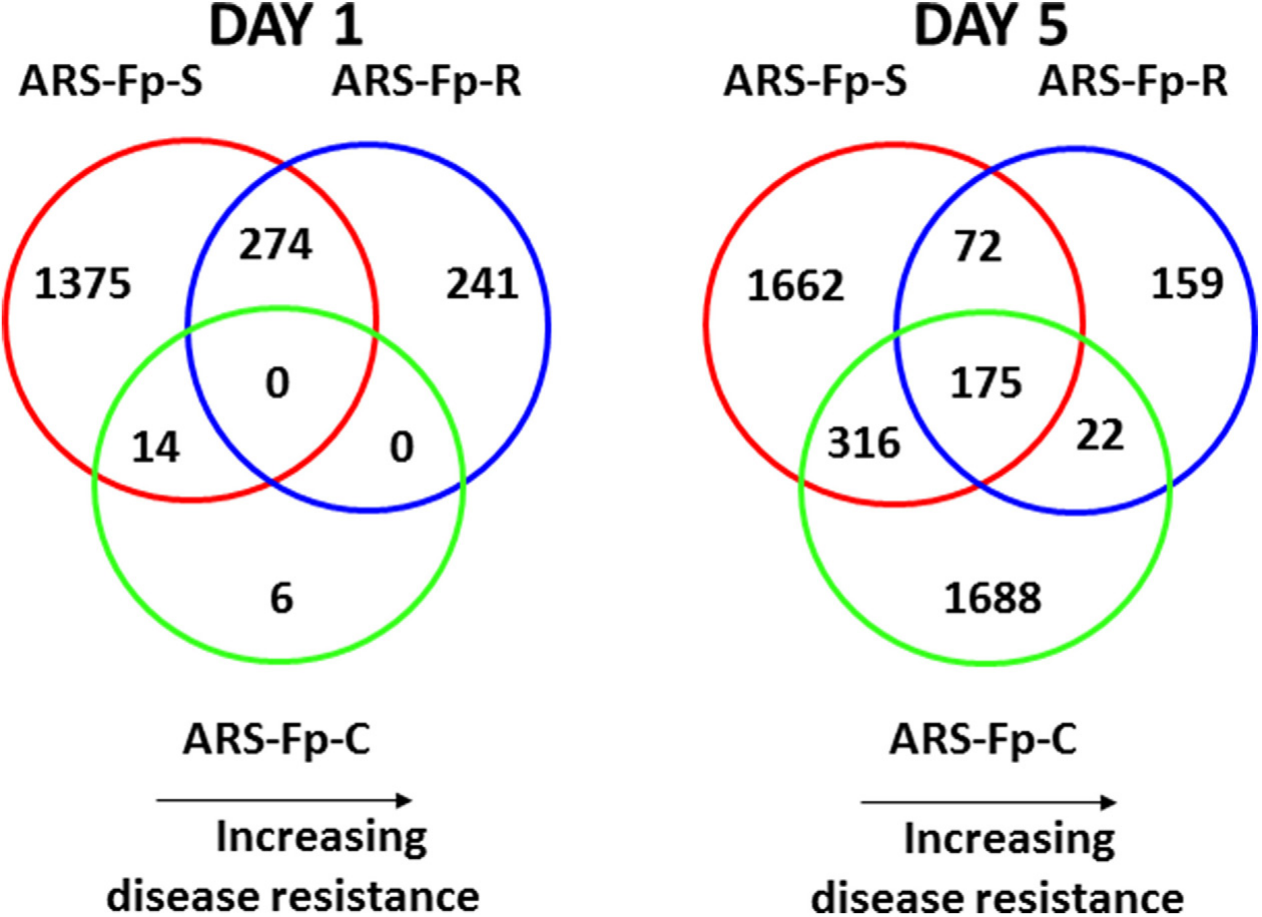
**Venn diagrams depicting commonalities of regulated genes in infected ARS-Fp-R, ARS-Fp-C, and ARS-Fp-S line fish that showed significant differences in transcript abundance compared to respective PBS-challenged fish groups on days 1 and 5 post-infection**. For all analyses, pair-wise comparisons were calculated with DESeq2 using a *q* < 0.05. Circles are color coded by line, ARS-Fp-S (red line), ARS-Fp-C (green), and ARS-Fp-R (blue).

**Figure 4:**
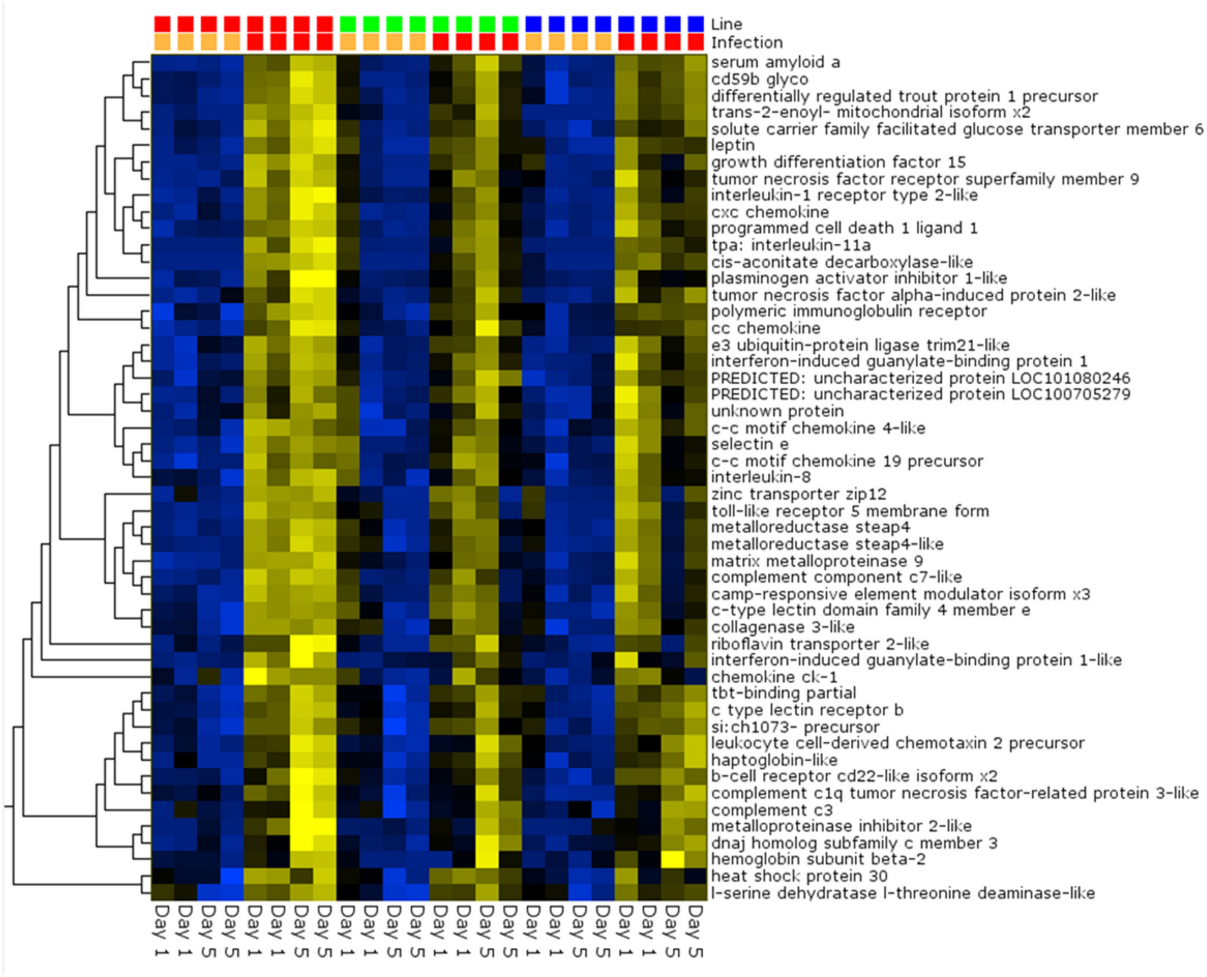
**Heat map of PCA analysis showing the most highly regulated genes in infected vs. day-matched PBS injected fish (*q* = 0.01 and >*log*_2_ 3-fold cut-off)**. Genetic line (S-line red color, C-line green color, R-line blue color) and infection status (PBS injected orange color, Fp injected red color) are shown on top and day post-infection is shown on bottom. Variables (genes) are grouped by hierarchal clustering.

Pair-wise analysis of differentially regulated genes shared between infected and PBS injected fish within each sampling day identified no common genes on day 1, although 274 were shared between ARS-Fp-R and ARS-Fp-S line fish (Figure 3). This may in part be due to the low numbers of genes differentially expressed between the ARS-Fp-C line replicates. Among all day five samples, 175 common genes were differentially regulated, by pair-wise comparisons, in all three lines (see Table S2, **Pair-wise** for the complete list). Of these, the majority (89%, *n* = 156) were upregulated, while only 19 were consistently downregulated. Application of stringent data filtering to the entire dataset (Qlucore, *q* = 0.01 and >3-fold *log*_2_ expression difference) identified 110 genes that were the most robust predictors of infection status that collapsed to 51 common sequence descriptions (Figure 4 and Table S2: **tab Qlucore *q* < 0.01, log_2_ > 3 fold** for complete gene list). Many shared coordinated patterns of gene expression across samples were identified by unsupervised hierarchal clustering (Figure 4). The most highly regulated immune relevant genes included serum amyloid A (GSONMT00016296001, GSONMT00005013001), complement c1q-like protein 2-like (GSONMT00002696001), differentially regulated trout protein 1 precursor (GSONMT00048193001, GSONMT00025517001), leukocyte cell-derived chemotaxin 2 precursor (GSONMT00024746001, GSONMT00065856001), toll-like receptor 5 membrane form (GSONMT00013855001), c-type lectin domain family 4 member e (GSONMT00005166001), c type lectin receptor b (GSONMT00023806001), cd59b glycoprotein (GSONMT00025518001), and interleukine-1 receptor type 2-like (GSONMT00066304001). Interestingly, a number of putative metabolic genes were also highly expressed in infected ARS-Fp-S line fish compared to the ARS-Fp-R line including cis-aconitate decarboxylase-like (GSONMT00057407001), tbt-binding partial (GSONMT00003889001), l-serine dehydratase l-threonine deaminase-like (GSONMT00025010001), leptin (GSONMT00002603001), and growth differentiation factor 15 (GSONMT00000024001), catechol o-methyltransferase domain-containing protein 1-like (3 genes) and hepcidin (GSONMT00082379001). Multiple paralogues of several less abundantly expressed immune relevant genes included complement component 3 (4 genes), interferon-induced guanylate-binding protein 1 (3 genes), interferon-induced guanylate-binding protein 1-like (5 genes), interferon-induced protein 44-like (3 genes) and microfibril-associated glycoprotein 4-like (3 genes). Differentially regulated cytokine genes included interleukin 11 (GSONMT00009406001) and interleukin 1-beta (GSONMT00005489001) as well as nine chemokine genes that included: cc chemokine (GSONMT00017873001, GSONMT00007278001), c-c motif chemokine 19 precursor (GSONMT00014841001, GSONMT00057082001), c-c motif chemokine 4-like (GSONMT00080018001), chemokine ck-1 (GSONMT00024124001), cxc chemokine (GSONMT00080684001), and interleukin 8 (GSONMT00038968001, GSONMT00059090001). Immune genes of interest also included, programmed cell death 1 ligand-1 (GSONMT00040812001), tnf receptor superfamily member 5 (GSONMT00012579001), tnf receptor superfamily member 6b (GSONMT00034343001) and tnf receptor superfamily member 9 (GSONMT00057532001), and b-cell receptor cd22-like isoform x2 (GSONMT00072668001).

### Gene expression in naive fish

Gene expression profiles were compared in PBS-injected ARS-Fp-R, ARS-Fp-C and ARS-FP-S line fish to examine the effect of selective breeding on the transcriptome of naive animals. In the first analysis, pair-wise comparisons between lines were examined. On day 1 post challenge, three genes demonstrated significantly different transcript abundance between PBS-injected ARS-Fp-R and ARS-Fp-C fish, 76 genes were different between the ARS-Fp-R and ARS-Fp-S line fish, and 28 genes were different between the ARS-Fp-C and ARS-Fp-S line fish (Table 2 and Supplementary Data Sheet 4: **tabs R_C PBS Day 1, R_S PBS Day 1, and S_C PBS Day 1, respectively**). None of these genes were common to all three comparisons and none were found to be similarly differentially regulated between genetic lines after challenge with *F. psychrophilum*. On day 5 post injection, the number of differentially regulated genes in naive fish increased with 246 genes demonstrating significant differences in transcript counts between the ARS-Fp-R and ARS-Fp-C lines, 45 genes between the ARS-Fp-R and ARS-Fp-S lines, and 61 genes between the ARS-Fp-C and ARS-Fp-S lines (Table 2). No genes were common to all three comparisons and eight genes exhibited a significant difference within two comparisons with similar trends in infected fish (Supplementary Data Sheet 4). Of these genes, only complement factor h-like (GSONMT00015052001) has a purported immunologic role. Fold gene expression differences were 2.8 and 2.1 between naive resistant and susceptible and infected resistant and susceptible fish, respectively. Complement factor h-like was 1.8 fold-higher in naive control fish compared to naive susceptible fish and 5.0 fold different between the infected lines (Supplementary Data Sheet 4).

**Figure 5:**
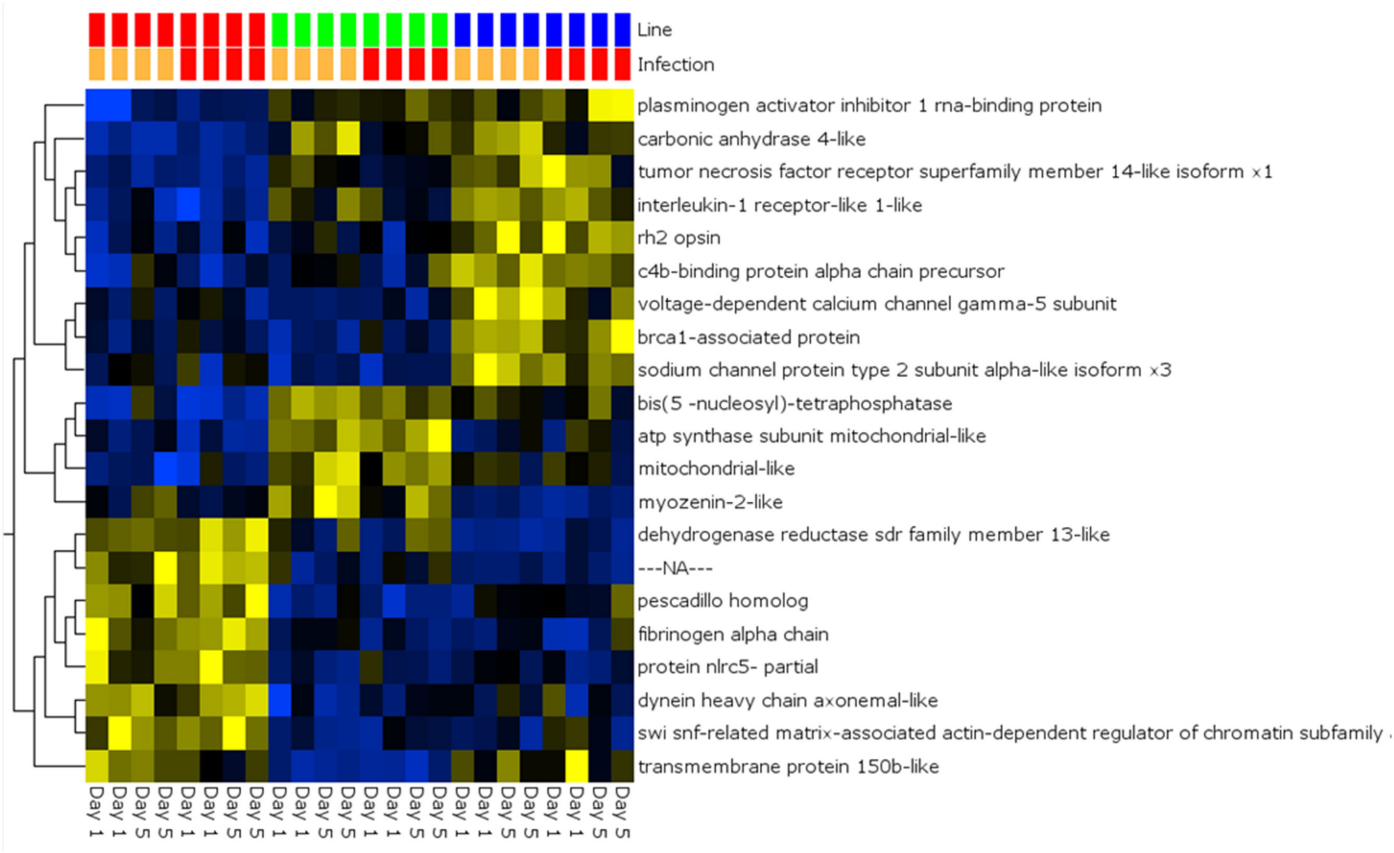
**Heat map showing multi-group comparison by genetic line eliminating infection as a factor (*q* = 0.05)**. Variables (genes) are grouped by hierarchal clustering.

**Figure 6:**
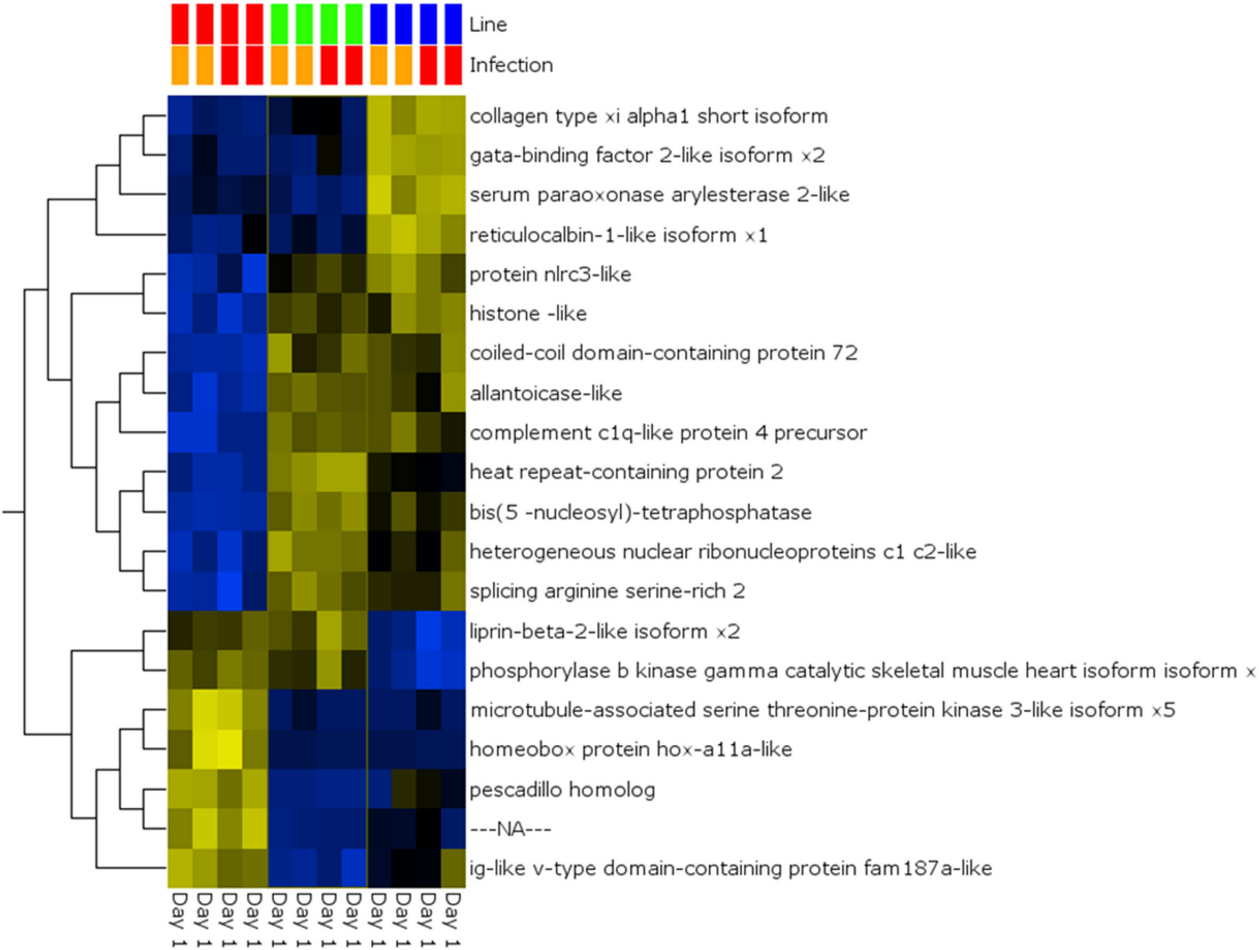
**Heat map showing multi-group comparison by genetic line eliminating infection as a factor (*q* = 0.05) for Day 1 samples**. Variables (genes) are grouped by hierarchal clustering.

A global analysis of expression differences between lines was performed on the entire dataset. In these analyses, data were first filtered for variance ≥2.5% and then subjected to *q* < 0.05 and infection status was removed as a factor. Within the complete dataset, 21 differentially expressed genes were identified (Figure 5). Several genes exhibited the pattern of low expression in ARS-Fp-S line, intermediate expression in ARS-Fp-C line and highest expression in ARS-Fp-R line. These included immune relevant genes tnf receptor superfamily member 14b-like isoform x1 and interleukine-1 receptor-like 1, genes. We also analyzed only day 1 samples, and identified additional immune relevant candidates that included complement c1q-like protein 4 precursor, protein nlrc3-like and gata-binding factor 2-like isoform x2 (Figure 6). Interestingly, there were also genes that exhibited the opposite expression pattern: higher in ARS-Fp-S line and lower in the ARS-Fp-C and ARS-Fp-R lines. This included putative immune relevant gene nlrc5-partial (Figure 5) and ig-like v-type domain-containing protein family 187a-like (Figure 6).

### Manual examination of known immune genes expressed in response to *F. psychrophilum* infection

In order to compare this dataset to previous analyses of gene expression following bacterial challenge, 23 known immune relevant genes were selected and *de novo* sequence alignment of the complete RNA-seq dataset was performed. There was a significant difference in transcript abundance between *F. psychrophilum* and PBS-challenged fish on day 1 and/or day 5 post-infection for 10/23 published gene sequence that were searched against the RNA-seq data set (Table 4) (*P_adj_* < 0.05). Seven genes demonstrated a significant difference in transcript counts between genetic lines on day 5 post-infection, with no significant differences occurring between genetic lines on day 1 (Table 4) (*P_adj_* < 0.05). There was no significant difference in transcript abundance between infected and control fish or between genetic lines for *tgf-β, tcr-β, sod1, mx-1, mt-a, mhc-I, il-6, il-4/13A*, and *foxp3b*. Transcript counts were too low to in at least one comparison to provide a significant *P_adj_*value for *tnf-α, il1- β, il-17c2*, and *inf-γ*.

**Table 4:** **Fold-change in transcript abundance of genes described as modulated by *Fp* challenge**.

**Annotation**	**Accession No**	***Fp* vs. PBS, Day1**			***Fp* vs. PBS, Day5**			***Fp* vs. *Fp*, Day5**		
		***R***	***C***	***S***	***R***	***C***	***S***	***R* vs. *S***	***R* vs. *C***	***C* vs. *S***
**PROINFLAMMATORY/ACUTE PHASE RESP**.										
*il-17-c1*	FM955453	3.9	–	17.2	8.8	3.6	41.8	–	–	–
*tlr5*	AB062504	–	–	8.0	2.6	2.1	18.4	0.4	–	–
*Saa*	AM422446	2.7	–	3.6	–	1.9	10.6	0.3	–	–
*il-8*	AJ279069	2.9	–	5.3	–	2.0	12.6	0.2	–	–
**ADAPTIVE PROCESSES**										
*cd3*	L24433	–	–	1.6	2.6	2.5	3.0	–	–	–
*cd4*	AY973028	–	–	–	1.9	2.4	1.7	–	–	–
*cd8*	AF178054	–	–	–	–	–	1.9	–	–	–
*Igm*	S63348	–	–	–	2.1	2.8	–	1.9	–	2
*Igt*	AY870265	–	–	–	2.1	2.8	–	1.9	–	–
*mhc-II*	AF115533	–	–	–	–	–	1.6	0.6	–	0.7

*Quantification was performed between Fp and PBS challenged ARS-Fp-R (R), ARS-Fp-C (C), and ARS-Fp-S (S) line fish and between genetic lines on days 1 and 5 post-infection. There were no significant differences between genetic lines on day 1 post-infection*.

### Validation of RNA-seq data by qPCR

**Figure 7:**
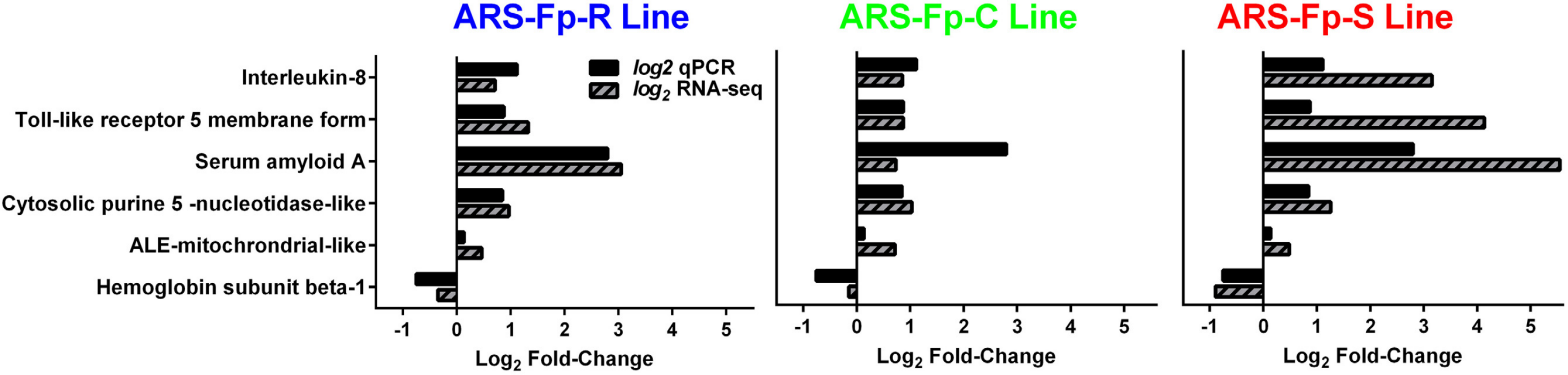
**Comparison between qPCR and RNA-seq by genetic line**. All samples were from day 5 post-challenge.

In order to begin validating RNA-seq transcript abundance estimates, selected differentially expressed genes were identified for qPCR analysis (Table S1). There was significant correlation between transcript fold-change values determined by RNA-seq and qPCR in control and infected fish on day 5 post-infection (*R* = 0.75, *P* < 0.001) (Figure 7).

## Discussion

To our knowledge this is the first study describing whole-body transcriptome analysis and comparison of three divergently selected lines of rainbow trout exhibiting graded survival differences in response to standardized *F. psychrophilum* challenge. We identified large numbers of differentially expressed genes between genetic lines that increased in number with time and bacterial load. Since we utilized whole fish in these experiments, we interpreted differences in transcript abundance as upregulation/downregulation of genes or alteration of transcript stability. Importantly, the use of entire fish for RNA extraction rules out the potential for cell migration to confound expression differences inherent in studies utilizing defined immune tissue source (i.e., spleen, blood, or anterior kidney). A limitation of our approach is the likelihood of missing differentially expressed genes expressed only in rare subsets of cells. Nevertheless, our depth of sequence coverage allowed quantification of transcript abundance from about 70.5% of all identified protein coding genes identified within the rainbow trout genome and approximately 68.2% of putative immune relevant genes that we identified by automated and manual annotation. Genes identified as differentially expressed were primarily associated with the acute phase response to bacterial infection but we also identified genes associated with innate and adaptive immune responses, physiologic and metabolic processes, and wound healing. This experimental design allowed for inclusion of both mucosal and systemic immune system tissue sampling and demonstrates previously unrecognized changes in gene transcription that occur with BCWD. These findings illustrate the high degree of transcriptional complexity involved in the rainbow trout BCWD response and provide a reference data set to begin to understand the impact of selective breeding on the genetic basis of disease resistance.

### Induction of the acute phase response and innate immunity

The transcriptional response included induction of complement factors, acute-phase proteins, cytokines, chemokines and other genes associated with innate immunity. A relatively high number of complement factors were identified as upregulated after infection. This included multiple transcript alignments to genes encoding complement c3, complement c9, complement c4-b-like, and complement c1q-like proteins. Generation of complement during the acute-phase response has been well-described in rainbow trout and likely imparts direct bactericidal activity (Sunyer and Lambris, 1998; Whyte, 2007). The magnitude and range of complement factor expression suggests the complement system constitutes an important aspect of the host response to *F. psychrophilum* although factors were not found to be significantly different between resistant and susceptible fish. A wide range of acute phase protein encoding genes, including serum amyloid A and tlr5 were identified as highly expressed after infection. Similar trends have been described from tissues of rainbow trout affected by BCWD (Overturf and LaPatra, 2006; Langevin et al., 2012). Differentially expressed trout protein is a relatively recently recognized immune factor shown to be expressed during the salmonid acute phase response after bacterial infection (Bayne et al., 2001; Tsoi et al., 2004). Along with IL-1, IL-11, and IL-17-c1, these factors likely exert diverse pro-inflammatory and defensive actions including recruitment of inflammatory cells and further amplification of the acute phase response (Stadnyk, 1994; Jorgensen et al., 2001; Carrington et al., 2004; Wang et al., 2009). Significantly higher transcript counts for acute phase proteins and cytokines in the ARS-Fp-S line on day 5 may represent higher induction of pro-inflammatory conditions compared to the ARS-Fp-R line that correlates with higher bacterial loads.

### Differential expression of genes that contribute to adaptive immunity

A limited number of differentially regulated genes were identified that may be associated with adaptive immune processes. There was modest up-regulation of *igm* and *igt* genes and cellular factors associated with cell signaling and activation including *mhc-II, cd3, cd4*, and *cd8* genes. Higher *igm* gene transcript levels in resistant and control-line fish compared to the susceptible-line suggest an earlier development of antibody mediated processes, although the converse pattern was observed for *mhc-II* gene transcript counts on the same days post-infection.

### Genes involved in wound healing

A number of genes associated with wound healing and wound response showed significant expression differences between naive and infected fish but no trends between genetic lines or sample day. These included syndecan-4-like isoform x2, plasminogen activator inhibitor 1-like, and ras-related c3 botulinum toxin substrate 2 precursor. All have roles in localized tissue repair in mammals through augmenting extracellular matrix reorganization, cellular growth and proliferation, and regulation of cell signaling (Romer et al., 1996; Woods and Couchman, 1998; Lin et al., 2005; Ojha et al., 2008). A limited number of genes with described roles in wound healing in other species (i.e., abhydrolase domain-containing protein 2-a-like and a collagen alpha-1 chain-like factor) demonstrated reduced expression in infected fish. Wound repair has not been well-characterized in fish and the general effect of these genes on the host response to infection is unknown. Necrosis of internal organs, peripheral skeletal muscle and skin is likely directly associated with host morbidity and mortality (Nilsen et al., 2011; Marancik et al., 2014b) and further studies are needed to determine how wound healing impacts recovery and survival.

### Identification of large paralogous families of putative immune-relevant genes

The recent rainbow trout genome contains expansion of putative gene families including protein nlrc3 and nlrc3-like (*n* = 111), protein nlrc5 (*n* = 9), polymeric immunoglobulin receptor-like (*n* = 14), perforin-1-like (*n* = 8), B-cell receptor cd22-like proteins (*n* = 38), cd209-like (*n* = 34), macrophage mannose receptor 1-like (*n* = 28), lrr and pyd domain-containing proteins (*n* = 37), and fish virus induced trim proteins (*n* = 44) (Supplementary Data Sheet 3). The availability of the genome allows a more comprehensive analysis of partially characterized immune gene families including tumor necrosis factor superfamily of ligands (*n* = 26) and toll-like receptors (*n* = 27) (Palti, 2011; Wiens and Glenney, 2011). Of note, a large number of tumor necrosis factor receptor superfamily members (tnfrsf, *n* = 59) were identified (Supplementary Data Sheet 3). While the precise phylogenetic nomenclature and grouping of tnfrsf remains, our analysis indicates many members are differentially regulated in response to *F. psychrophilum* infection including tnfrsf 1a precursor (GSONMT00019008001), tnfrsf 1a-like (GSONMT00061996001) tnfrsf 5 precursor (GSONMT00012579001, GSONMT00003531001), tnfrsf 5-like (GSONMT00013182001), tnfrsf 6 (GSONMT00082555001), tnfrsf 6b (GSONMT00034343001), tnfrsf 6b-like (GSONMT00020936001), tnfrsf 9 (GSONMT00057532001), tnfrsf 9-like (GSONMT00050654001), tnfrsf 19-like isoform x1 (GSONMT00055755001) and tnfrsf 19-like isoform x2 (GSONMT00069336001). In addition, basal expression of one paralogue of tnfrsf 14-like isoform x1 (GSONMT00000915001, from a total *n* = 11 paralogues present in the genome) is modestly higher in the ARS-Fp-R line as compared to the ARS-Fp-S line (Figure 5). While Blast2GO v.2 provides a description for novel sequences based on natural language text mining functionality (Gotz et al., 2008), we emphasize that detailed annotation and phylogenic analysis of these genes remains to be undertaken. Some of these genes may be pseudogenes and it is likely that the sequence description of many of the automated annotations we present here will require revision following expert curation, and may also change as improvements are made to the reference rainbow trout genome sequence and analysis of transcript variants.

### Differentially expressed genes between naive fish from the three genetic lines

There was tight clustering of data and a low number of gene expression differences between PBS-injected fish. This suggests phenotypic differences between the ARS-Fp-R, -C, and -S lines are largely induced by infection and that selective breeding appears to have had a relatively low impact on basal gene expression during the normal physiologic state. Expression of complement factor-h like was observed to be significantly different between both naive and infected resistant and susceptible fish. Complement factor-h is a regulatory protein of the alternative complement pathway and although isolated from rainbow trout (Anastasiou et al., 2011), its contribution to the rainbow trout immune response has not yet been characterized.

### Comparison of differentially regulated genes with Langevin et al. study

This study expands on the work of Langevin et al. (2012) who described differential regulation of select genes by microarray and qPCR in the anterior kidney of BCWD resistant and susceptible rainbow trout clones after experimental infection. Both studies showed an increase in transcription of genes encoding pro-inflammatory cytokines, anti-bacterial effectors and matrix metalloproteases. Notably, complement factors, serum amyloid A, mannose-binding proteins, chemokines, and interleukins 1 and 8 all showed significant transcriptional increase on day 5 post-infection. RNA-seq data provides further evidence for upregulation of immunorelevant factors not previously identified, including tlr5, leptin, haptoglobin, and C-type lectin and genes associated with adaptive, physiologic, structural, and intracellular process. There was no evidence to support differential regulation of interferon-gamma during infection in either study although potential interferon induced genes are differentially regulated. Tumor necrosis factor-alpha was also notably absent from both studies despite previously published (Evenhuis and Cleveland, 2012) and unpublished data (Wiens, unpublished data) suggesting upregulation during infection. Assay sensitivity may have been confounded by whole-body RNA isolation which could reduce the ability to detect low abundant cytokines expressed in specific tissues.

There was variability between studies when transcriptomic responses were compared between resistant and susceptible line fish. Both studies observed higher bacterial loads in susceptible fish and increased transcriptional response of a number of genes associated with pro-inflammatory conditions including interleukin-1, cc chemokine and matrix metalloproteinases 1 and 19. Our study further identified serum amyloid A, differentially regulated trout protein, and cytochrome p450 1a as significantly upregulated in susceptible fish. A number of metalloproteinase inhibitors were identified by Langevin et al. (2012) as significantly upregulated in resistant but not susceptible fish but showed no significant differences between genetic lines when analyzed in our study. This variability likely extends from apparent differences in experimental design including challenge route, bacterial dose and strain, tissue sampled, and microarray vs transcript count quantification and analysis. However, even with these differences in method, both studies demonstrated a robust immune response in *F. psychrophilum* challenged fish with significant differences in the transcriptome of resistant and susceptible fish, associated with immune relevant genes.

## Concluding remarks

Complex transcriptional differences were identified between lines following infection with *F. psychrophilum* strain CSF259-93. Most of the differentially regulated genes exhibited increased transcript abundance and correlated with higher bacterial loads. It is likely that most of the changes we identified are consequences of the differential expansion of *F. psychrophilum* following infection, especially in the ARS-Fp-S line. However, differences in bacterial load cannot account for transcriptional differences observed on day 1, as bacterial loads were similar between genetic lines. Future efforts will be directed at dissecting early time points following exposure and will focus on identifying inter-individual differences as this study examined pools of fish due to sequencing cost limitations. This data will be integrated with proteomic studies to examine the relationship between transcript and protein levels and to assist in exploring biomarkers of infection, immunocompetency and disease resistance. Also, in this study we did not analyze long non-coding RNA, microRNA and splice variants. Despite these limitations, we suggest that this data set represents an important future resource for exploration of candidate genes identified from QTL analyses being conducted in parallel with this study. Quantitative trait loci (QTL) mapping has identified nine QTL on seven chromosomes that have moderate to large effects on resistance (Vallejo et al., 2014a). We have focused on an Omy19 QTL (Wiens et al., 2013b) and confirmed inheritance in a subsequent generation (Vallejo et al., 2014b). By combining chromosomal position known for many of the genes within this dataset, we have begun to identify differentially regulated genes present on Omy19 and other chromosomes as proof of principal for this approach. Further studies are needed to validate and fine-map the BCWD QTL and these studies are currently underway. These complex patterns support a polygenetic architecture of BCWD resistance and will serve as a reference dataset for identifying mechanisms associated with the genetic basis of disease resistance.

### Conflict of interest statement

The authors declare that the research was conducted in the absence of any commercial or financial relationships that could be construed as a potential conflict of interest.
